# Hyaluronan-CD44 Interaction Promotes Growth of Decidual Stromal Cells in Human First-Trimester Pregnancy

**DOI:** 10.1371/journal.pone.0074812

**Published:** 2013-09-19

**Authors:** Rui Zhu, Song-Cun Wang, Chan Sun, Yu Tao, Hai-Lan Piao, Xiao-Qiu Wang, Mei-Rong Du, Da-Jin Li

**Affiliations:** 1 Laboratory for Reproductive Immunology, Hospital and Institute of Obstetrics and Gynecology, IBS, Fudan University Shanghai Medical College, Shanghai, China; 2 Department of Obstetrics and Gynecology, the First Affiliated Hospital of Soochow University, Suzhou, China; Virgen Macarena University Hospital, School of Medicine, Spain

## Abstract

Hyaluronan (HA) and its receptor CD44 are expressed at the maternal-fetal interface, but its role in early pregnancy remains unclear. Here, we found that primary decidual stromal cells (DSCs) continuously secreted HA and expressed its receptor CD44. Pregnancy-associated hormones up-regulated HA synthetase (HAS) 2 transcription and HA release from DSCs. High molecular weight-HA (HMW-HA), but not medium molecular weight (MMW-HA) or low molecular weight (LMW-HA), promoted proliferation and inhibited apoptosis of DSCs in a CD44-dependent manner. The in-cell Western analysis revealed HMW-HA activated PI3K/AKT and mitogen-activated protein kinase (MAPK)/ERK1/2 signaling pathways time-dependently. Blocking these pathways by specific inhibitor LY294002 or U0126 abrogated HMW-HA-regulated DSc proliferation and apoptosis. Finally, we have found that HA content, HA molecular weight, HAS2 mRNA level, and CD44 expression were significantly decreased in DSCs from unexplained miscarriage compared with the normal pregnancy. Collectively, our results indicate that higher level and greater molecular mass of HA at maternal-fetal interface contributes to DSc growth and maintenance of DSCs in human early pregnancy.

## Introduction

Decidual stromal cells (DSCs) are the major cellular component at the maternal–fetal interface, comprising 75% of decidual cells, and are particularly important owing to their pleiotropic functions during pregnancy. In addition to their traditional nutrition and support to embryo in pregnancy, growing evidence suggests that DSCs are involved in immune modulation, including antigen phagocytosis and presentation, followed by cytokine production [1,2,3]. Moreover, DSCs are the main source of extracellular matrix (ECM) components that mediate extravillous trophoblast (EVT) invasion and homeostatic protection during trophoblast invasion [4,5,6,7], and serve as sensors of embryo quality upon implantation [8]. DSCs substantially modulate peripheral immune cell infiltration into the decidua [9,10]. Although DSCs are an important cell type at the maternal-fetal interface, their biological function in early pregnancy remains almost unclear.

DSCs are differentiated from fibroblast-like precursor cells in the decidual tissue. Differentiation of endometrial stromal cells into decidual stromal cells, decidualization, is critical for embryo implantation and pregnancy establishment. A defective decidualization is associated with recurrent pregnancy loss [11,12]. Pregnancy is characterized by high levels of sex steroid hormones which regulate the coordinate progression of decidualization, placentation, and embryo development [13,14].

Hyaluronan (HA) is a non-sulfated glycosaminoglycan polymer of repeating disaccharide units of N-acetylglucosamine and β-glucuronic acid. HA is a prominent component of ECM, particularly in rapidly growing and remodeling tissues. HA is synthesized by three different HA synthetases (HASs), namely HAS1, HAS2, and HAS3, but HAS2 is responsible for the synthesis of high-molecular-weight hyaluronan (HMW-HA) and is involved in a variety of cellular functions including proliferation, differentiation, and inflammation [15]. The transmembrane glycoprotein, CD44, is the predominant receptor for HA on cell surface. Binding of HA to CD44 has been implicated in lymphocyte homing, tumorigenesis, and monocyte activation [16]. HA has a strong negative charge, and the large water volume of hydration associated with HA causes hydration and expansion of tissues, thus creating an environment that permits cell proliferation.

Both HA and CD44 are observed in the early human conceptus and in decidual stroma [17,18]. Hyaluronan-enriched transfer medium significantly increased pregnancy and implantation rates in patients with multiple embryo transfer failures, suggesting that HA is essential for embryo implantation and pregnancy [19]. However, little is known about the role of HA-CD44 interaction in the biological behaviors of DSCs at the maternal-fetal interface. In the present study, we analyzed HA and CD44 expression in human DSCs of the first-trimester gestation, and investigated the regulation of the pregnancy-related hormones on HA and HA synthetases. We further investigated the role of HA-CD44 interaction in the behavior of DSCs and their intracellular signaling pathways. Finally, we compared HA and CD44 expression in DSCs between the normal early pregnancy and unexplained miscarriage.

## Materials and Methods

### Reagents

The following pregnancy-associated hormones were used to stimulate DSCs: 0.001-100 nM estradiol; 0.001-100 nM progesterone; or 1.25-10.0 KU/ml human chorionic gondaotropin (hCG, Sigma-Aldrich, Inc., St. Louis, MO, USA). Different weight of HA (15-40kDa, 75-350 kDa, and >950KDa, i.e., low, medium, and high molecular weight; LMW-HA, MMW-HA, and HMW-HA, respectively) were obtained from R&D system. Biotinylated hynaluronan-binding protein (bHABP) was from Sigma-Aldrich. HA-binding antagonistic peptide PEP-1 (H2N-GAHWQFNALTVR-OH) and scrambled control peptide (H2N-WRHGEALTAVNQ-OH) were obtained from Invitrogen™ (Life Technologies Corp., Grand Island, NY). Anti-CD44 neutralizing antibody (Clone 5F12) was from Thermo, Fisher (Scientific Inc., Fremont, CA, USA). PE conjugated anti-human Vimentin and APC conjugated anti-human CD44 antibody were from Biolegend, USA. PI3K/AKT signal pathway inhibitor LY294002 and MAPK/ERK1/2 signal pathway kinase (MEK1/2) inhibitor U0126 were purchased from Cell Signal Technology. Primary antibodies for In-cell Western: rabbit polyclonal anti-Akt, anti-ERK1/2 (Santa Cruz Biotechnology, USA), mouse monoclonal anti-phospho-Akt (Thr308/309/305), and anti-phospho-ERK1/2 (Tyr204/202) (Santa Cruz Biotechnology, USA).

### Human decidual tissue collection

The first-trimester human decidual tissues were obtained from 28 healthy women in early pregnancy (28.46±5.23 years old of 53.5±5.68 gestational days, mean±SD), the pregnancy was terminated for non-medical reason. Decidual samples were obtained from 11 spontaneous abortions during the first trimester of pregnancy (28.81±5.19 years old at 52.55±6.5 gestational days, mean±SD). All the normal pregnancy and miscarriage were confirmed by ultrasound. All women were not on medication, nonsmokers, and with a history of regular menstrual cycles. The counterpart placenta of each decidua presented normal chromosome. The decidual samples from miscarriage were excluded from inflammation and necrosis. All tissues were immediately collected into ice-cold DMEM/F12 tissue culture medium (Gibco Inc, Grand Island, NY, USA), transported to the laboratory within 30 min after surgery, and washed in calcium- and magnesium-free Hanks balanced salt solution (HBSS) for DSC isolation. All procedures involving study participants were approved by the Human Research Ethics Committee of the Obstetrics and Gynecology Hospital, Fudan University, China, and all the subjects provided written informed consent for the collection and use of the tissue samples.

### Isolation and primary culture of DSCs

DSCs were isolated by collagenase type IV (Sigma-Aldrich, USA) digestion and discontinuous Percoll gradient centrifugation, as described previously [3,6,7]. DSCs ranging in density between 1.042-1.062 g/mL were cultured in DMEM/F12 supplemented with 10% fetal bovine serum (FBS, Invitrogen), 100 U/mL penicillin, and 100 µg/mL streptomycin (complete medium) in 5% CO_2_ at 37°C. After culture for 30 min, the non-adherent lymphocytes were rinsed off, making DSC >95% pure by characterization of the primary DSCs [6]. The purity of DSCs was confirmed by immunocytochemistry and flow cytometry. The primary DSCs from normal early pregnancy were seeded in 6-, 12-, 24-well plates with 1×10^6^, 5×10^5^, 2×10^5^ cells per well in 1.0 ml complete medium, respectively. After 12, 24, 36, 48, 60, 72h, the supernatants were collected for HA content determination. The primary DSCs from unexplained miscarriage were seeded into 6-well plates at 1×10^6^ cells/1.0 ml/well for 72 h, and the supernatants were harvested for HA content determination. For hormone addition, the primary DSCs from normal early pregnancy were seeded into 24-well plates at 2×10^5^ cells/1.0ml/well for 72 h after treatment with the pregnancy-associated hormones. At the indicated time points following hormone addition, supernatants were quantified for HA.

### Immunostaining

The decidual tissues were processed for immunohistochemical staining, sections were incubated with mouse anti–human cytokeratin 7 (CK-7, 1:100), vimentin (1:50) (Santa Cruz Biotechnology, USA) antibodies and rabbit anti-human CD44 antibody (clone EPR1013Y, 1:50, Epitomics, USA) or mouse and rabbit isotype-matched IgG overnight at 4°C in a humidified chamber, followed by incubation with a biotinylated goat anti-mouse or anti-rabbit secondary antibody. To detect the HA expression in decidual tissues, bHABP(10 µg/mL) was used. The slides were then incubated with the avidin-biotin–horseradish peroxidase complex, and were stained with diaminobenzidene and counterstained with hematoxylin. The specificity of the staining of HA was determined by preincubating tissue samples with 10 U/mL Streptomyces hyaluronidase (HYAL, Sigma-Aldrich) at 37°C for 2 hours in a humidification chamber and then staining with bHABP. All images were captured by Olympus BX51 microscopy. Original magnification was ×400 for all panels.

### Immunofluorescence

After 24 hour of culture, the primary DSCs were washed and fixed with phenolformaldehyde (4%; 20 min, room temperature), washed with PBS and permeabilized (10 min, room tempreture) with 0.2% Triton X-100 in PBS. Samples were blocked with 2% BSA in PBS followed by incubation (overnight, 4°C) with the primary Abs. Rabbit anti-human vimentin mAb (Cell Signalling, 1:50) was used as markers for identification of DSC, bHABP (10µg/ml) was used as detection of HA expression. Isotype-matched irrelevant IgG (Sino-America) was used as a control. After incubation with primary Ab, the cells were washed with PBS-0.1% Tween 20, and then incubated with fluorescein-isothiocyanate (FITC)-conjugated avidin and phycoerythrin (PE)-conjugated rabbit secondary Abs. After washing, DAPI nuclear stain was added to the cells, which were then washed and mounted with Vectashied (Vector, Burlingame, CA, USA). Florescence images were captured by Leitz DMRX microscope. The experiments were repeated three times.

### HA content determination

HA concentration in DSC supernatants was measured with a competitive ELISA by using bHABP as described previously [20]. Briefly, samples and bHABP were incubated in a microtube for 1 h. The sample-bHABP mixtures were added into hyaluronan-coated microtiter Nunc CovaLink™ NH modules (Thermo Fisher Scientific, Inc.). Bound bHABP were measured with a colorimetric reaction A standard curve was generated with hyaluronan of known concentration (range 0–2000 ng/ml).

### HA synthetase (HAS) mRNA quantification by real-time reverse transcription PCR

Real-time RT-PCR was performed as our previous methods [21]. To compare the HAS mRNA level in the DSC cells, the following primers were used: HAS1, forward: 5’-GCC TCA GTT TCC CTC CTC TG-3’, reverse: 5’-CCT TTC CCT CCA CTC CTC AG-3’; HAS2, forward: 5’-GCC TCA TCT GTG GAG ATG GT-3’, reverse: 5’-TCC CAG AGG TCC ACT AAT GC-3’; HAS3, forward: 5’-GGC ATT ATC AAG GCC ACC TA-3’, reverse: GAC ACA GGA ATG AGG CCA AT; human β-actin, forward: 5’-CTA CGT CGC CCT GGA CTT CGA GC-3’; reverse: 5’-GAT GGA GCC GCC GAT CCA CAC GG-3’.

### Cell proliferation assay

The freshly isolated DSCs were seeded at 2×10^4^ in flat-bottomed 96-well culture plates overnight. The media was replaced with DMEM/F12 for 12 h, and then treated with complete media containing different molecular weight HA (100 µg/ml), HA antagonist and control peptides (100 µg/ml) with or without pretreatment of neutralizing anti-CD44 antibody (20 µg/ml) or PI3K signal pathway inhibitor LY294002 (50 µM) or MEK1/2 inhibitor U0126 (30 µM) for 30 min, then cultured at 37°C for 48 h. respectively. Cell proliferation was analyzed with a BrdU Proliferation Assay kit using manufacturer protocol (Calbiochem® Biochemicals, San Diego, CA, USA).

### Annexin V and propidium iodide (PI) staining for cell apoptosis

The freshly isolated DSCs were seeded at 2×10^5^ cells/well in 24-well plates overnight, and were treated as above. The cells were harvested and resuspended in 100 µl annexin-binding buffer with 5 µl FITC-annexin V and 1 µl PI working solution (BD Bioscience), and then were incubated in the dark for 15 min at room temperature, and additional 400µl binding buffer was added, and DSCs were analyzed immediately by flow cytometry (BD Biosciences, Franklin Lakes, NJ).

### In-cell Western Assay for expression of the signal molecules in DSCs

According to the description by Egorina [22] and our previous procedure [6], a newly set-up assay called in-cell Western was taken to determine the in-cell protein level of Akt, ERK1/2, and the phosphorylated Akt and ERK1/2. DSCs cultured in 96-well tissue culture plates were stimulated with HMW-HA for different time (0, 30, 60, 90, 120 minutes), and were immediately fixed with 4% paraformaldehyde for 20min at room temperature. The cells were then washed with 0.1% Triton, followed by blocking with 150µl of LI-COR Odyssey Blocking Buffer (LI-COR Biosciences, Lincoln, Nebraska, USA) for 90min at room temperature, and incubated with rabbit anti-human Akt (1:50), rabbit anti human ERK1/2 (1:50), mouse anti-human phospho-Akt (1:50) or mouse anti-human phospho-ERK1/2 (1:50) antibodies. After incubation at 4°C overnight, the wells were washed and incubated with corresponding second IRDyeTM700DX-conjugated affinity purified (red fluorescence) anti-mouse antibody and IRDyeTM800DX-conjugated affinity purified (green fluorescence) anti-rabbit antibody at the recommended concentration (Rockland, Inc., Gilbertsville, PA, USA) in the dark for 60 min. Images of the target gene were obtained by using the Odyssey Infrared Imaging System (LI-COR Biosciences German version of Ltd.). The expression level of the protein molecules was calculated as the ratio of the intensity of phosphoresed protein to correspondent total proteins. The experiments were carried out in triplicate, and repeated three times.

### HA molecular weight determination

To determine the molecular size distribution of HA produced by DSCs, the polyacrylamide gel electrophoresis was carried out [23,24]. 1×10^6^ DSCs from normal early pregnancy and miscarriage were seeded in 100-mm dishes, respectively. Cells were cultured for 3 days after reaching confluence and then changed to fresh serum-free medium. 72-hour supernatants were collected for experiments. The supernatant collected from DSCs was concentrated with centrifugal filter (10,000-d cut-off; Millipore, Billerica, Mass) and then digested with protease Pronase (100 U/mL, Pronase from *Streptomyces griseus*, Calbiochem) at 55°C for 2 hours, followed by inactivation of protease activity by boiling the samples at 100°C for 10 minutes. Concentrated samples along with known molecular mass hyaluronan standards (15-40 kDa, 75-350 kDa, and >950KDa, R&D) were electrophoresed on a 4-20% concentration gradient of polyacrylamide(Bio-rad, USA), stained with 0.005% Stain-All (Sigma, St Louis, Mo) dissolved in 50% ethanol solution in the dark for 1 h, and then destained in 10% ethanol solution for 2 h with constant shaking, and final destaining was completed by exposing the gel to amber light for 10 minutes. Hyaluronan-polyacrylamide gels were photographed on an Odyssey Infra-red Imaging System (LI-COR Biosciences German version of Ltd.). Quantitative analysis of the calibrated image was accomplished using ImageJ software. A standard curve was determined by using known molecular mass HA standards. HA peaks in the samples were calculated against the standard curve.

### Statistical analysis

Statistical comparisons were performed by using one-way analysis of variance (ANOVA) or two-way ANOVA or t test and least significant difference (equal variances assumed) or Tamhane’s test (equal variances not assumed) or unpaired t test with SPSS software version 15.0 (Chicago, IL, USA). All error bars in figures indicate standard error (SE). Statistical significance was accepted at P <0.05. 

## Results

### 1: DSCs express HA and its receptor CD44 in human early pregnancy

**Figure 1:**
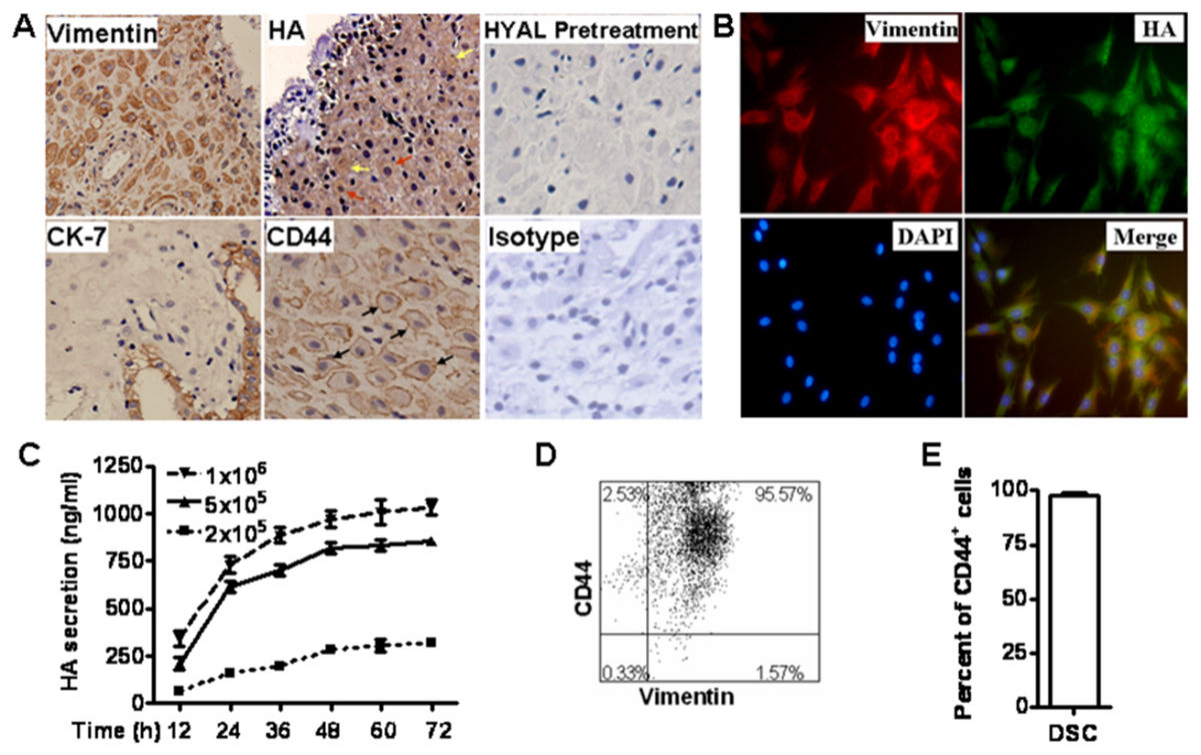
Expression of HA and its receptor CD44 in DSCs from human early pregnancy. HA and CD44 expression was confirmed by immunohistochemistry, immunofluorescence, ELISA and flow cytometry, respectively. (A) The decidual tissues from normal early pregnancy were stained for HA and CD44. Slides were pretreated with HYAL to determine the specificity of HA. The red and yellow arrows showed HA staining in the cytoplasm and extracellular matrix, respectively (C) Primary DSC cells were seeded at 2×10^5^, 5×10^5^, or 1×10^6^ cells/ml in cell culture plates. After 12, 24, 36, 48, 60 and 72 h of culture, the supernatants were collected and measured by ELISA. Each point represents the mean ± SE of triplicate values from four independent experiments. (D, E) The primary DSCs were stained by CD44 and vimentin, and the percentage of CD44^+^Vimentin^+^ DSC cells was analyzed by flow cytometry (FCM). The flow cytometric picture is from three different experiments.

The immunohistochemistry was used to detect the expression of HA and CD44 in the decidual tissues from normal early pregnancy. As shown in Figure 1A, DSCs are characterized by vimentin positive and CK7 negative in the cytoplasm, while decidual gland epithelial cells (DECs) are positive with CK7 and negative with vimentin. The cytoplasm of DSCs (red arrows) and extracellular matrix (yellow arrows) were positively stained by HA, and the membrane of these cells were positive with CD44 (black arrows) (Figure 1A).

After 24 h of culture, we characterized DSCs by using immunofluorescence. As shown in Figure 1B, DSCs were stained by vimentin and HA with red or green color in the cytoplasm, respectively. The cell nucleus appeared blue after staining by DAPI. After merging, the cytoplasm of all the cells was orange, suggesting that vimentin ^+^ DSCs expressed HA in their cytoplasm which presents orange color.

DSCs had a strong proliferative ability and a long survival period in vitro. When seeded on cell-cultured plate, they can survive for more than 1 week. The primary DSCs cultured at different densities were examined every 12 h by ELISA for soluble HA release into the supernatant. Figure 1C shows that the secretion of HA was correlated to cell density, and increased continuously during the 72 h culture period. HA levels in DSC supernatants increased most rapidly during the first 24 hours. During 48-72 hours the HA levels reached the plateau and no more rise in the concentrations. Accumulated HA concentrations were 1,032.43±73.83 ng/ml, 878.94±19.47 ng/ml, and 319.15±26.06 ng/ml, when seeded at 1×10^6^ cells/ml, 5×10^5^ cells/ml and 2×10^5^ cells/ml, respectively, for 72 h. Flow cytometry showed that >95% of the DSCs expressed HA receptor, CD44 (Figure 1D, 1E).

### 2: Pregnancy-associated hormones promote DSCs secreting HA

**Figure 2:**
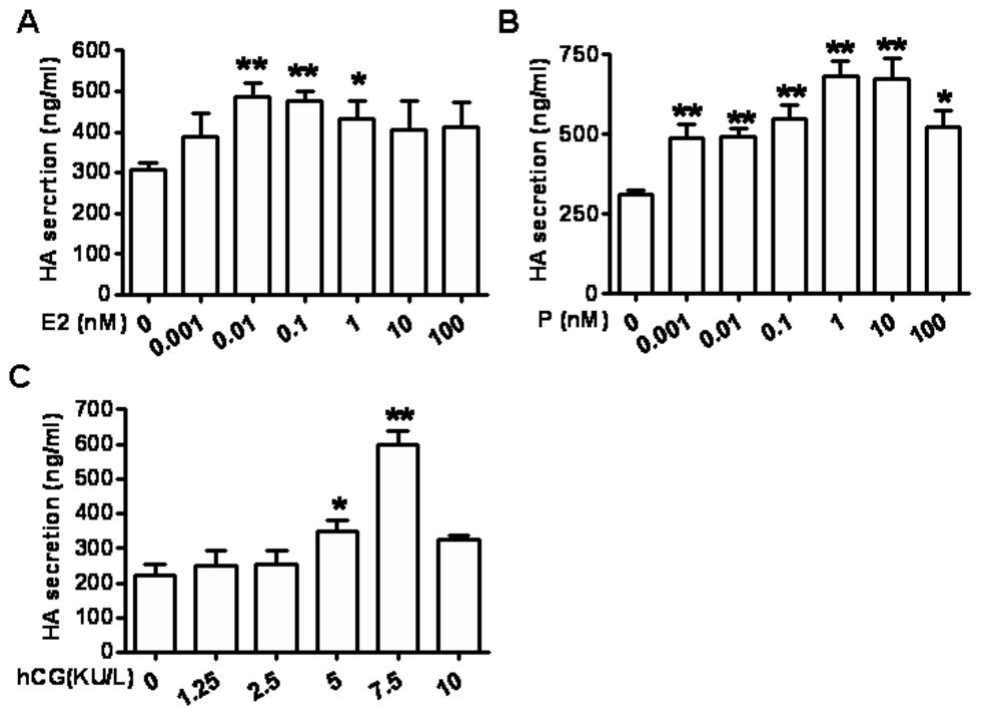
Pregnancy-associate hormones promote HA secretion from DSCs. DSCs were seeded at 2×10^5^ cells/ml in cell culture plates and were stimulated with different concentrations of estradiol (A), progesterone (B), hCG (C) for 72 h. Supernatants were harvested and measured for HA by ELISA. Data are the mean±SE of triplicate values from three independent experiments. *P<0.05, **P<0.01, compared to the control.

We investigated whether the pregnancy-related hormones could regulate HA secretion by human DSCs. The DSCs were treated with different concentrations of corresponding hormones and different combinations of these hormones for 72 hours, and then supernatants were analyzed for HA by ELISA. As shown in Figure 2A, estradiol up-regulated HA secretion in a concentration-related manner. The increased HA release was approximately 1.5-fold at concentrations of 0.01-1.0 nM compared to the vehicle control, and maximal induction occurred at 0.01 nM, declining to baseline at 10 nM (Figure 2A). Progesterone and hCG promoted HA secretion by DSCs in a similar biphasic concentration-associated manner. HA secretion peaked in 1.0 nM progesterone and 7.5 KU/L hCG, respectively, and declined to baseline levels at higher hormone concentrations (Figure 2B, 2C). These data indicate that the physiological concentrations of hormones increase HA secretion of DSCs. However, the combinations of two or three sorts of hormones did not enlarge the promotion of HA secretion by DSCs induced by any hormone alone (Data not shown).

### 3: Pregnancy-associated hormones enhance HAS mRNA transcription in DSCs

**Figure 3:**
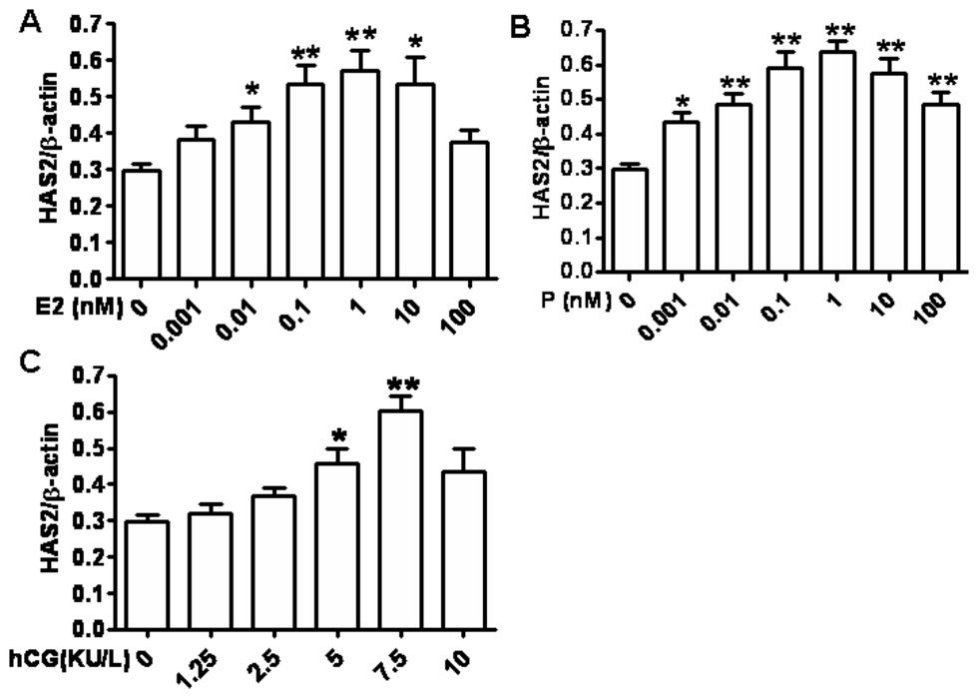
Pregnancy-associated hormones enhance HAS mRNA transcription of DSCs. DSCs were seeded at 5×10^5^ cells/ml in cell culture plates and were stimulated with different concentrations of estradiol (A), progesterone (B), hCG (C) for 24 h. The cells were harvested and subjected to RNA extraction and real-time PCR for detection of mRNA levels of HAS1, HAS2, and HAS3. Data are the mean±SE of triplicate values from three independent experiments. *P<0.05, **P<0.01, compared to the control.

We next determined whether the pregnancy-associated hormones promoted HA secretion by up-regulation of HAS. After treated with different concentrations of indicated hormones for 24 hours, real-time PCR was used to determine mRNA levels of three HASs of primary DSCs. The results showed that estradiol, progesterone and hCG up-regulated HAS2 mRNA level of DSCs in concentration-related manner. HAS2 mRNA level peaked in 1.0 nM estradiol, 1.0 nM progesterone and 7.5 KU/L hCG, respectively (Figure 3A, 3B, 3C). Unexpectedly, neither HAS1 nor HAS3 mRNA could be detected in DSCs after 40 cycles whatever cultured alone or treated with pregnancy-associated hormones, indicating that HAS1 and HAS2 were almost unexpressed in DSCs. Furthermore, the combinations of hormones didn’t show the synergetic promotive effects on HAS2 mRNA level in DSCs (Data not shown).

### 4: HMW-HA promotes growth of DSCs via binding to CD44

**Figure 4:**
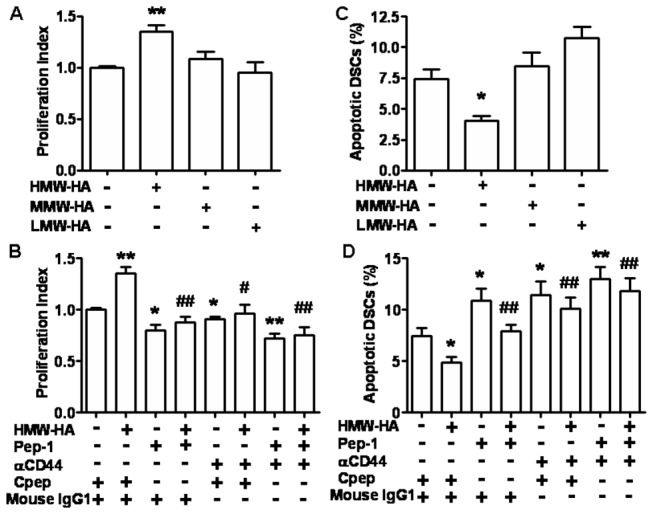
HMW-HA promotes growth of DSCs via binding to CD44. The primary DSCs in 96-well plates or 24-well plates were treated with different sizes of HA for 48 h. BrdU assay (A, B) and flow cytometry (C, D) were used to analyze DSC proliferation and apoptosis, respectively. The proliferation index of DSC cells under different conditions was normalized to the unstimulated control. Apoptotic cells were defined as AnnexinV ^+^ PI^-^ DSCs. Control, isotype control IgG; HA, 100 µg/ml; α-CD44, anti-CD44 neutralizing antibody 20 µg/ml; Cpep, control HA peptide, 100 µg/ml; Pep-1, HA antagonist peptide, 100 µg/ml. Data represent the mean±SE of four separate experiments. *P<0.05, **P<0.01, compared to the control; ^#^ *P*<0.05, ^# #^ *P*<0.01, compared to HMW-HA treatment.

We then examined the effects of different molecular weight HA on the proliferation and apoptosis of human first-trimester DSCs. As shown in Figure 4A, HMW-HA, but not MMW-HA or LMW-HA, promoted human DSC proliferation. The addition of HA antagonist peptide completely abolished the stimulatory effect of the exogenously administrated HMW-HA on DSC cell proliferation. Moreover, blocking HA-CD44 interaction by anti-CD44 neutralizing antibody abrogated the HMA-HA-promoted proliferation. Meanwhile, the basal proliferation of DSCs was also inhibited by treatment with HA antagonist peptide or anti-CD44 neutralizing antibody (Figure 4B), which suggests that endogenous and exogenous HMW-HA promotes proliferation of DSCs via binding to CD44 in an autocrine manner. As expected, treatment with HMW-HA inhibited DSC apoptosis (Figure 4C). Similarly, the addition of HA antagonist peptide or anti-CD44 neutralizing antibody completely eliminated the apoptosis inhibition of DSC by HMW-HA. If DSCs were treated with the HA antagonist peptide or anti-CD44 neutralizing antibody, DSC apoptosis was significantly higher than the control (Figure 4D). However, combination of HA antagonist peptide and anti-CD44 neutralizing antibody showed no synergistic effect on DSC cell proliferation and apoptosis regulated by HMW-HA (Figure 4B, 4D).

### 5: PI3K/AKT and MAPK/ERK1/2 pathways are involved in the HA/CD44-regulated DSC proliferation and apoptosis

**Figure 5:**
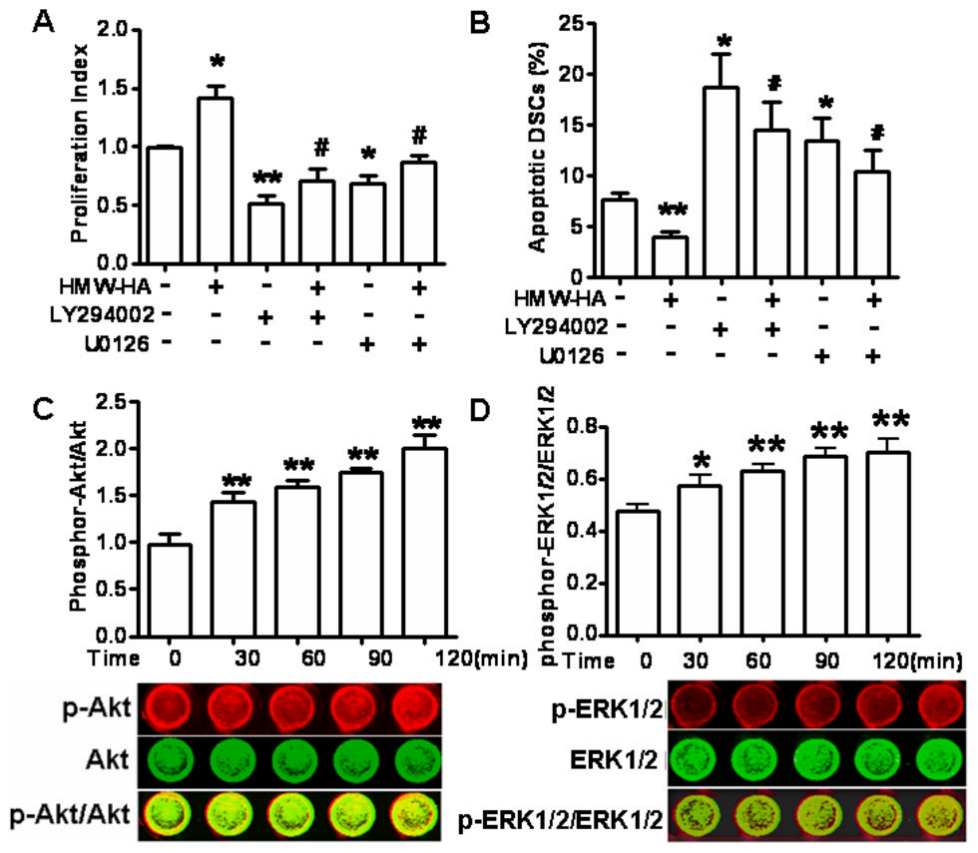
PI3K/AKT and MAPK/ERK1/2 pathways are involved in the HA/CD44-regulated DSC proliferation and apoptosis. The DSCs were treated for 48 h with either 0.1% DMSO (control/vehicle), 100 µg/ml HMW-HA, 50 µM PI3K/AKT inhibitor LY294002, 30 µM MEK1/2 inhibitor U0126, or 100 µg/ml HWW-HA combined with 50µM LY294002 or 30µM U0126. BrdU proliferation assay (A) and flow cytometry (B) were used to detect the proliferation and apoptosis, respectively. In-cell Western (C, D) HMW-HA activated in vitro PI3K/AKT and MAPK/ERK1/2 of DSC cells. The primary-cultured DSCs were starved with FBS-free media for 12 h and then treated with 100 µg/ml HMW-HA for different time. The levels of phosphor- and total- AKT and ERK1/2 were determined by In-cell Western. The pictures were from the typical one of three individual experiments. The red represents phospho-AKT or ERK1/2 and the green represents total- AKT or ERK1/2. Data represent the mean±SE of three experiments performed in triplicate wells. *P≤0.05, **P≤0.01 compared to the control; ^#^ *P*<0.05, compared to HMW-HA treatment.

The PI3K/AKT and MAPK/ERK1/2 signaling pathways represent downstream targets of activated HA-CD44 interaction [25,26,27]. We therefore investigated these signaling pathways in mediating HMW-HA-regulated DSC proliferation and apoptosis. As shown in Figure 5A, HMW-HA up-regulated proliferation and these effects were completely abrogated by the PI3K/Akt inhibitor LY294002 and the MEK1/2 inhibitor U0126, and the proliferation index was even lower than that of the untreated (Figure 5A). Similarly, pretreatment with LY294002 or U0126 could completely reverse the apoptosis inhibited by HMW-HA. The percentage of apoptotic cells was even higher than that of the control (Figure 5B). These findings indicate that HMW-HA can promote growth of human DSCs through the PI3K/Akt and MAPK/ERK1/2 signaling pathways. The Figure 5A and 5B also showed that PI3K/AKT and MEK1/2 inhibitors exerted an important effect on basal proliferation and apoptosis of DSC. Based on this finding, we next analyzed AKT and ERK1/2 activation in DSC cells treated with HMW-HA by In-cell Western using phospho-specific antibodies. As shown in Figure 5C and 5D, after being serum-starved for 12 h, phosphorylation of AKT and ERK1/2 increased significantly upon administration of HMW-HA for 30 min or longer. The peak of AKT and ERK1/2 phosphorylation lasted up to 120 min. The analysis of lysates for the total expression of AKT and ERK1/2 ensured the equal loading of proteins in different lanes.

### 6: Differential expression of HA, HAS2 and CD44 in DSCs from normal pregnancy and miscarriage

**Figure 6:**
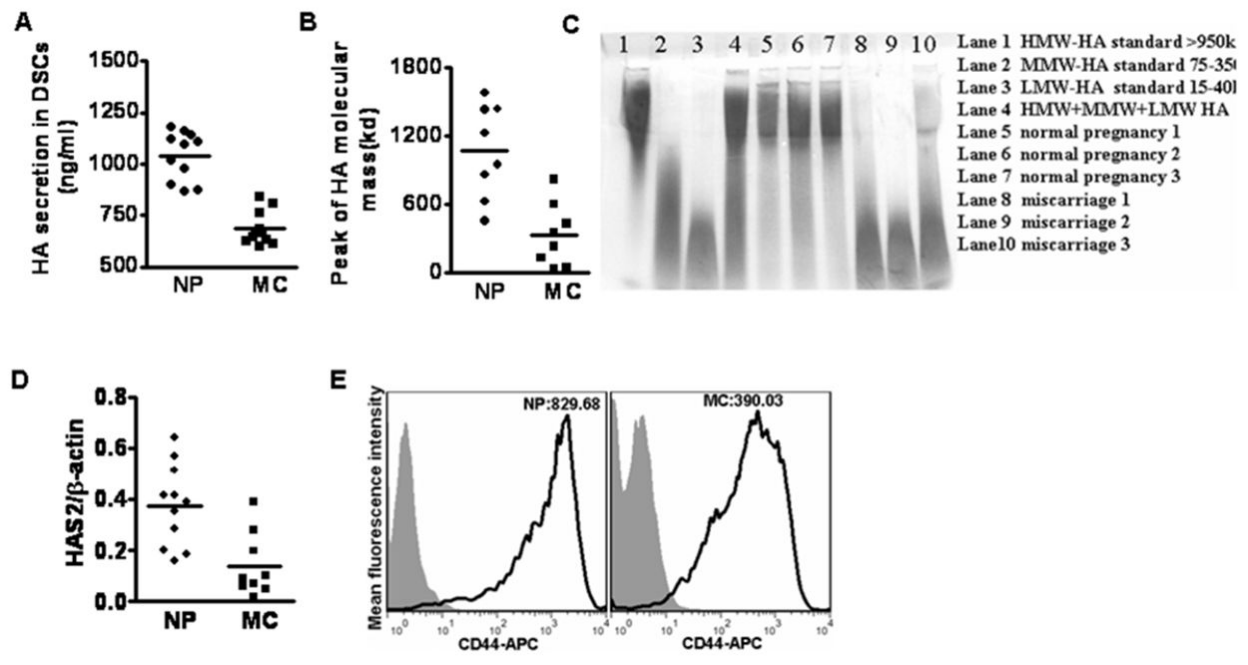
Differential expression of HA, HAS2 and CD44 in DSCs from normal pregnancy and unexplained miscarriage. (A) Primary DSCs from normal pregnancy and unexplained miscarriage were seeded at 1×10^6^ cells/ml in cell culture plates and cultured for 72h. The DSC supernatants were collected and measured by ELISA. Each dot represents results from different sample. (B, C) Molecular size of HA produced by DSCs from normal pregnancy and unexplained miscarriages was displayed through electrophoresis on polyacrylamide gels. Histogram showed the relative molecular mass of HA as determined by using ImageJ software. Data represent the mean ±SE of three independent experiments with a total of 8 samples from unexplained miscarriage and 8 samples from normal early pregnancy. (C) is one representative picture. (D) Primary DSCs from normal pregnancy and unexplained miscarriage were obtained and subjected to real time RT-PCR. HAS2 mRNA level in DSCs was compared between normal pregnancy and unexplained miscarriage. HAS2 mRNA level in DSCs from normal pregnancy was higher than that from unexplained miscarriage. (E) FCM analysis of the expression of CD44 on DSCs from normal and miscarriage. Data are mean ± SE from 4 repeated experiments, respectively. NP is normal pregnancy, and MC is unexplained miscarriage.

We first measured HA secretion by DSC cells from the early normal pregnancy or unexplained miscarriage. It was found that HA secretion by DSCs from the healthy early pregnancy was significantly higher (1039.18±35.52ng/ml) than that of miscarriage (684.83±24.65ng/ml) (Figure 6A). Culture supernatant of DSCs were concentrated and electrophoresed on polyacrylamide gels to determine the molecular mass distribution of HA. We found that DSCs from patients with miscarriage produced LMW-HA compared with the normal pregnancy (Figure 6B, 6C). Furthermore, the HAS2 mRNA level in DSCs from the normal pregnancy was much higher than that of miscarriage (Figure 6D). Although there was no significant difference on the percentage of CD44-positive DSCs from the normal pregnancy and miscarriage, the mean fluorescence intensity of CD44 on DSCs from miscarriage was significantly decreased (Figure 6E).

## Discussion

**Figure 7:**
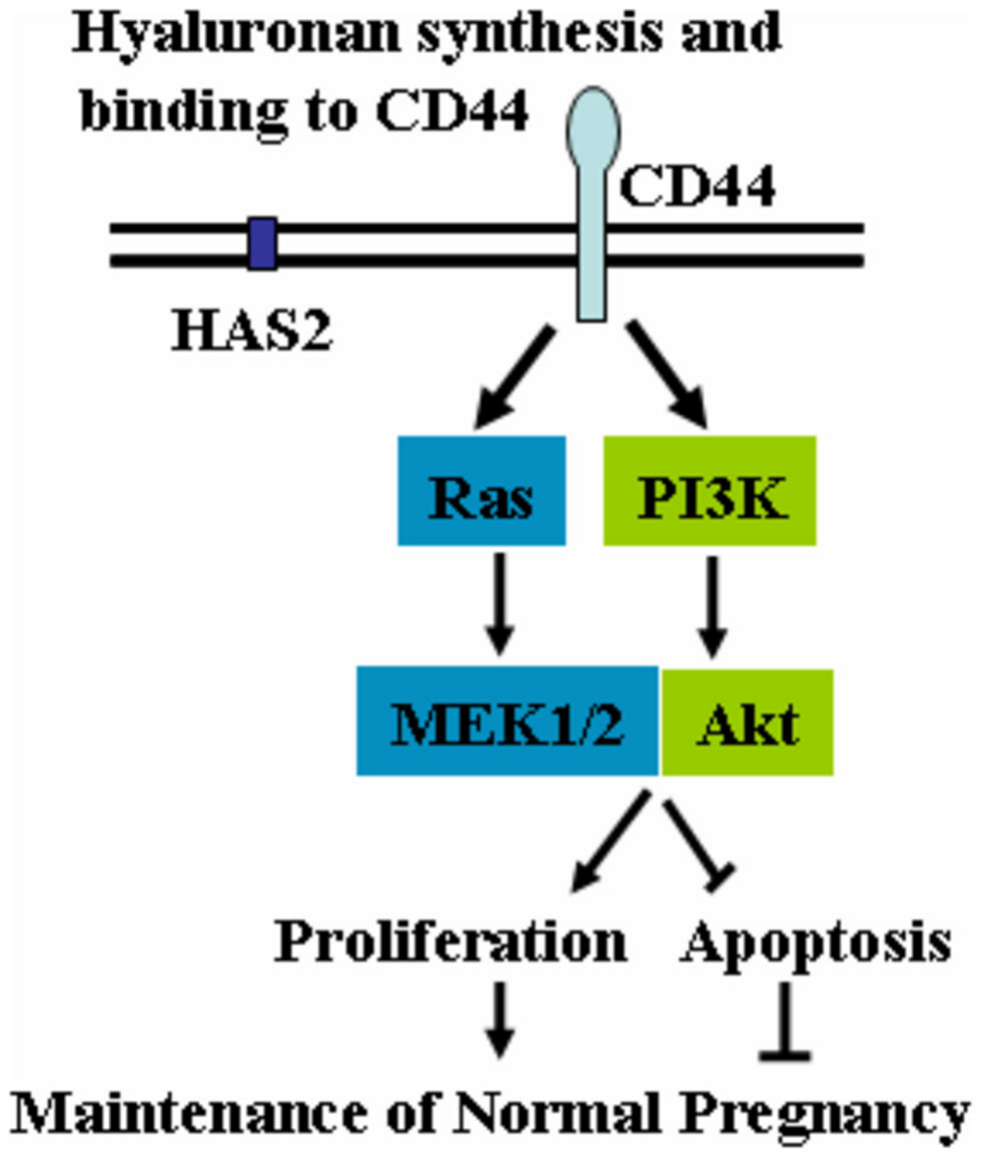
A proposed model for the role of HA in growth of DSCs in human early pregnancy. DSCs from normal pregnancy express higher HAS2, which synthesize higher molecular size of HA. HMW-HA activates PI3K/Akt and MAPK/ERK1/2 signalings via binding to CD44 in DSC membrane and then promotes cell proliferation while inhibits cell apoptosis, which is beneficial to normal pregnancy maintenance.

In this study, we have explored the roles of HA and its predominant receptor CD44 in the DSC biological behaviors during human early pregnancy. Our results indicate that DSCs from normal early pregnancy continuously secret high concentrations of HMW-HA and express its major receptor CD44. HMW-HA–CD44 interaction promotes DSC proliferation while inhibits DSC apoptosis via PI3K/Akt and MAPK/ERK1/2 signaling pathways. In addition, DSCs from women with unexplained miscarriage showed decreased HA production, its synthetase HAS2 and CD44 expression, leading to impaired HMW-HA-CD44 interaction and adverse pregnant outcome (Figure 7)

DSCs are the major cellular component of the maternal uterine decidua, into which the allogeneic fetus-derived trophoblast deeply invades. DSCs are endowed with important nutritive, endocrine capacities, and operate together with trophoblasts, modulating trophoblast invasion and placentation [6]. As a non-professional antigen presentation cells (APCs), DSCs play a crucial role in the regulation of decidual CD4^+^ T-cell cytokine production and help maintain a balanced cytokine milieu at the maternal-fetal interface [28]. As the main ECM-producing cells in the decidua, DSCs are regulated by their matrix environment where sex hormones are enriched. Our present data have also demonstrated that hormones at the maternal–fetal interface might play critical roles in maintenance of normal pregnancy via regulating HA production of DSCs.

HA biosynthesis is carried out at the inner face of the plasma membrane by HAS and the growing polymer is extruded through the membrane into the extracellular space. Three different HASs (HAS1, HAS2 and HAS3) have been isolated and characterized in mammalian cells. It was reported that HAS1 and HAS3 generated HA with broad size distributions (molecular masses of 2 x10^5^ to ~2 x 10^6^ Da), whereas HAS2 was the major synthetase of HA with a broad but extremely large size (average molecular mass of >2 x 106 Da), so HAS2 is responsible for the synthesis of HMW-HA [15,29]. HA exerts its biological roles via specific cell surface receptors by forming co-receptor complexes with various receptor tyrosine kinases. We have demonstrated that both HA and its predominant receptor, CD44, were expressed in the early human DSCs. The interaction of HMW-HA and CD44 contributes to DSC growth that has been verified by the fact that HMW-HA promoted DSC proliferation and inhibited DSC apoptosis which was abrogated by either pretreatment of HA antagonist peptide or anti-CD44 neutralizing antibody. Furthermore, there was no significant difference of DSC proliferation and apoptosis among the pretreatment of HA antagonist peptide, anti-CD44 neutralizing antibody and their combination. These data indicated that CD44 but not other HA receptors was essential in the regulation of HA on DSC growth.

The pregnancy-associated hormones including estrogen, progesterone, and hCG stimulate HAS2 transcription and HA production by DSCs. This is in agreement with the findings that estrogen specifically and preferentially promotes HA expression in skin cells [30] and hCG induces HAS2 and HA in murine granular cells during the ovulatory process [31]. Hormone-induced HA secretion of DSCs was dose-associated, peaking at a physiological level observed in human early pregnancy. Interestingly, the secretion of HA and CD44 expression by DSCs from normal pregnancy were much higher than that of unexplained miscarriage. More importantly, HA secreted by DSCs from normal pregnancy were mainly higher molecular mass while DSCs derived from miscarriage produced LMW-HA. Furthermore, the mRNA level of HAS2, not HAS1 or HAS3, was much higher in DSCs from normal pregnancy than that from miscarriage, similar with the fact that HAS2 is responsible for the synthesis of HMW-HA. Our results suggested that higher level of HMW-HA at the maternal-fetal interface maintained successful pregnancy, relative lower molecular weight of HA might contribute to pregnancy wastage. Our study is distinct from the previous report that enhanced hyaluronan expression and abnormal localization at the fetomaternal interface might be associated with murine abortion [32]. The discrepancy might be due to the different species and different detection method we investigated. Our results are accordance with the several reports that HMW-HA-enriched transfer medium increases implantation rate and improves outcome in cleavage-stage frozen-thawed embryo transfers and in patients with multiple embryo transfer failures [19,33]. Consistently, HMW-HA, but not MMW-HA or LMW-HA, promoted DSC proliferation. Similarly HMW-HA, but not MMW-HA or LMW-HA prohibited DSC apoptosis. However LWM-HA treatment increased DSC apoptosis. These data suggest that HA displays different biological roles depending on molecule weight. HA weight is critical for HA action [16,34,35,36]. This is keeping with the report that HMW-HA inhibits apoptosis while HA oligosaccharides induce apoptosis in tumor cell lines [37]. However, the molecular mechanisms of this size-dependent HA actions remain unknown. Different molecule weight of HA might exert their biological roles through binding to different receptors. HMW-HA functions via interaction with CD44 while hyaluronan fragments acted as an endogenous danger signal by engaging toll-like receptors [24,34,38,39]. The modulation of different molecular size of HA interacting with other receptors on DSC functional behaviors will be involved in our future study.

Increasing evidence suggests that CD44 is a critical mediator of both growth factor- and HA-induced mitogenic and invasive signaling in cancer cells [40,41]. HA-CD44 interaction promotes lymphocytic leukemia cell survival by activating PI3K/AKT and MAKP/ERK1/2 [25,26,27]. HA facilitates TGF-β1-dependent fibroblast proliferation through promoting interaction of CD44 with EGFR, which then induces cell proliferation via MAPK/ERK1/2 activation [39,41]. As demonstrated by the observation that HMW-HA activates PI3K/Akt and MAPK/ERK1/2 time-dependently, HMW-HA up-regulates proliferation and down-regulates apoptosis, and these effects are markedly abrogated by the PI3K/Akt inhibitor LY294002, or the MEK1/2 inhibitor U0126. Our findings validate that PI3K/Akt and MAPK/ERK1/2 signal pathways are involved in the regulation of HA-CD44 on DSC cells.

In summary, we have demonstrated the expression and function of HA/CD44 of human DSCs in the first trimester of pregnancy, and HA promotes DSC proliferation and growth in an autocrine manner. The high level and mature state of HA favor maintaining normal pregnancy, whereas lower content and degradation of HA may lead to early pregnancy wastage. Our study also shows that a physiological level of the pregnant-associated hormones including estrogen, progesterone, and hCG stimulate HAS2 transcription, which is important for HA production by DSCs in normal pregnancy. In pregnancy failure, the decreased hormones might not be sufficient to stimulate DSCs to express HAS2, resulting in deficient hyaluronan synthesis and DSC function. Therefore, our findings shed new light on the role of HA/CD44 in decidualization and placentation in human early pregnancy. Targeting the regulation of DSCs and extracellular matrix may present a novel therapeutic strategy in improving the outcome of pregnancy.
